# Supplementary material for: Discovery of clinically relevant fusions in pediatric cancer
Source: BMC Genomics. 2021 Dec 4;22:872. doi: 10.1186/s12864-021-08094-z (PMC8642973; doi:10.1186/s12864-021-08094-z)
Supplement: Supplementary file 1 — Additional file 1. [file 12864_2021_8094_MOESM1_ESM.pdf]

-- ADDITIONAL FILE 1: SUPPLEMENTAL FIGURES AND TABLES --

DISCOVERY OF CLINICALLY RELEVANT FUSIONS IN PEDIATRIC CANCER

Stephanie LaHaye[1], James R. Fitch[1], Kyle J. Voytovich[1], Adam C. Herman[1], Benjamin J. Kelly[1], Grant E. Lammi[1], Jeremy Arbesfeld[1], Saranga Wijeratne[1], Samuel J. Franklin[1], Kathleen M. Schieffer[1], Natalie Bir[1], Sean D. McGrath[1], Anthony R. Miller[1], Amy Wetzel[1], Katherine E. Miller[1], Tracy A. Bedrosian[1], Kristen Leraas[1], Elizabeth A Varga[1], Kristy Lee[1], Ajay Gupta[2], Bhuvana Setty[2,3], Daniel R. Boué[4,5], Jeffrey R. Leonard[3,6], Jonathan L. Finlay[2,3], Mohamed S. Abdelbaki[2,3], Diana S. Osorio[2,3], Selene C. Koo[4,5], Daniel C. Koboldt[1], Alex H. Wagner [1,3,7], Ann-Kathrin Eisfeld[8-10], Krzysztof Mrózek[9,10], Vincent Magrini[1,3], Catherine E. Cottrell[1,3,4], Elaine R. Mardis[1,3], Richard K. Wilson[1,3], Peter White[1,3,*]

[1] The Steve and Cindy Rasmussen Institute for Genomic Medicine, Nationwide Children's Hospital, Columbus, OH. [2]Division of Hematology, Oncology, Blood and Marrow Transplant, Nationwide Children's Hospital, Columbus, OH. [3]Department of Pediatrics, The Ohio State University, Columbus, OH. [4]Department of Pathology, The Ohio State University, Columbus, OH. [5]Department of Pathology, Nationwide Children's Hospital, Columbus, OH. [6]Section of Neurosurgery, Nationwide Children's Hospital Columbus, OH. [7]Department of Biomedical Informatics, The Ohio State University, Columbus, OH. [8]Division of Hematology, The Ohio State University, Columbus, OH. [9]Clara D. Bloomfield Center for Leukemia Outcomes Research, The Ohio State University, Columbus, OH. [10]The Ohio State Comprehensive Cancer Center, Columbus, OH.

***Corresponding Author:** Prof. Peter White, PhD, The Steve and Cindy Rasmussen Institute for Genomic Medicine, Nationwide Children's Hospital, 575 Children's Crossroad, Columbus, OH 43215. USA. Tel: +1 (614) 355-2671; Email: peter.white@nationwidechildrens.org

Keywords: transcriptomics, genomics, pediatric neoplasms, gene fusions, cancer, RNA-Seq

Running title: Fusion Identification in Pediatric Cancer

FIGURE S1

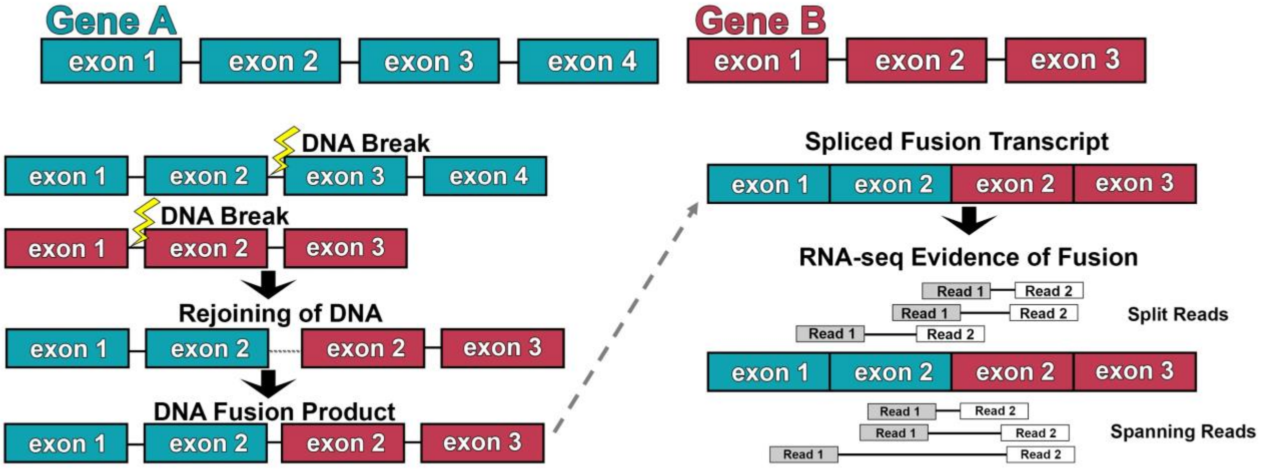

Figure S1. Fusion occurrence and identification by RNA-Seq. Fusions occur due to structural changes in genomic DNA, resulting in fusion transcripts. Gene A (blue) and Gene B (red) undergo DNA breaks, rejoin, and create a fusion product. This fusion product undergoes splicing and generates a fusion transcript, which can be identified through RNA-Seq technology. Paired end RNA-Seq identifies fusions through split reads, in which one read includes the junction of the fusion, or through spanning reads, where read 1 (gray) and read 2 (white) map to different genes.

FIGURE S2

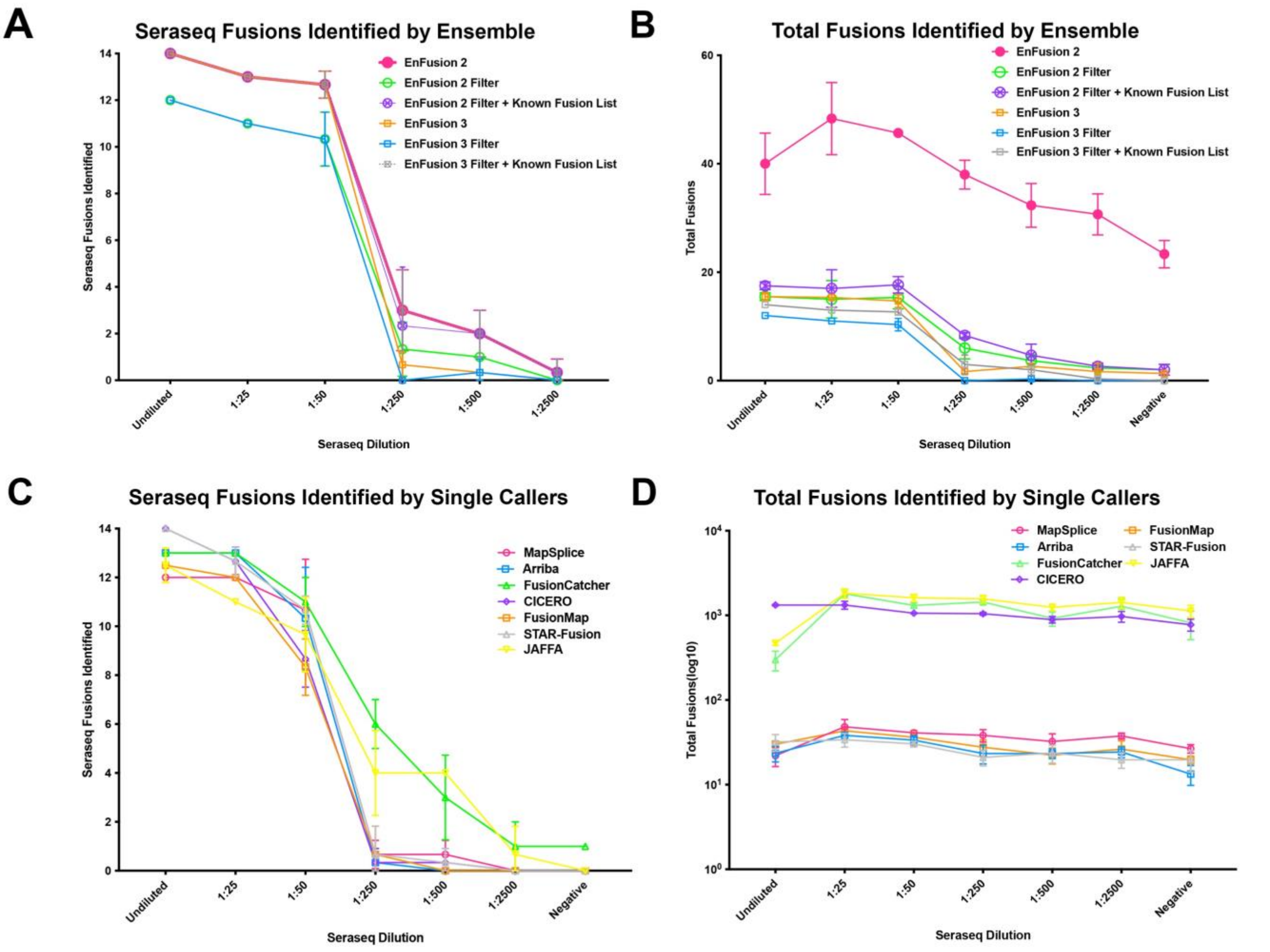

Figure S2. Limit of detection comparison of ensemble approach and single fusion detection algorithms. A) Seraseq fusions and **B)** total fusions identified by ensemble approach, including EnFusion ≥ 2 callers (EnFusion 2), EnFusion ≥ 2 callers with filtering (EnFusion 2 Filter), EnFusion ≥ 2 callers with filtering and known fusion list (EnFusion 2 Filter + Known Fusion List), EnFusion ≥ 3 callers (EnFusion 3), EnFusion ≥ 3 callers with filtering (EnFusion 3 Filter), and EnFusion ≥ 3 callers with filtering and known fusion list (EnFusion 3 Filter + Known Fusion List). **C)** Seraseq fusions and **D)** total fusions identified by each individual caller. Total fusions shown on log10 scale.

FIGURE S3

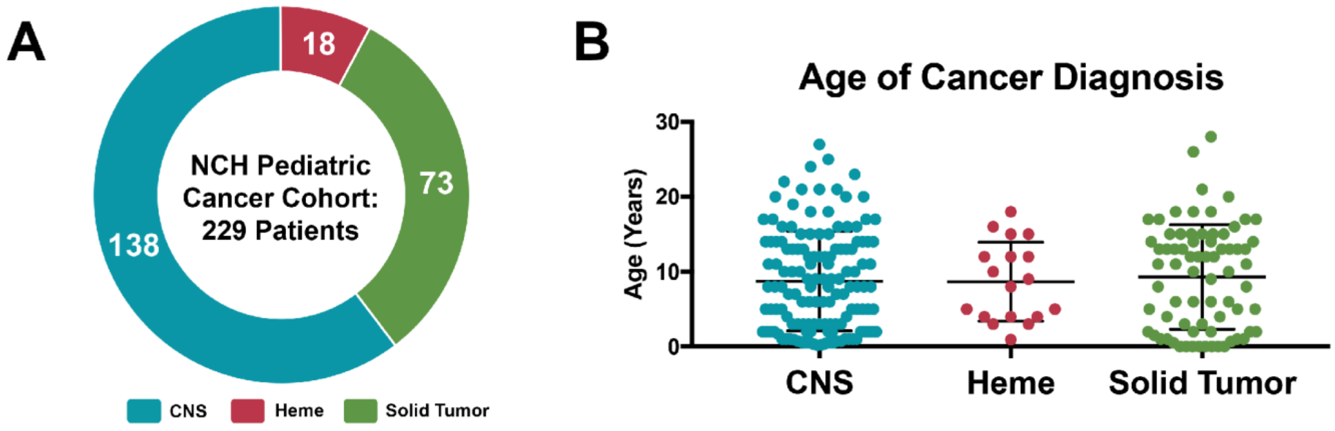

Figure S3. Pediatric cancer and hematologic disease cohort at Nationwide Children's Hospital. A) The Nationwide Children's Hospital (NCH) pediatric cancer and hematologic disease cohort, includes three IRB approved studies with a total of 229 patients, consisting of 138 Central Nervous System (CNS) tumors (blue), 18 Hematologic Diseases (Heme; red), and 73 Solid Tumors (green). **B)** The average age of cancer diagnosis for patients included in this study was 8.9 years and is shown broken down by tumor type.

FIGURE S4

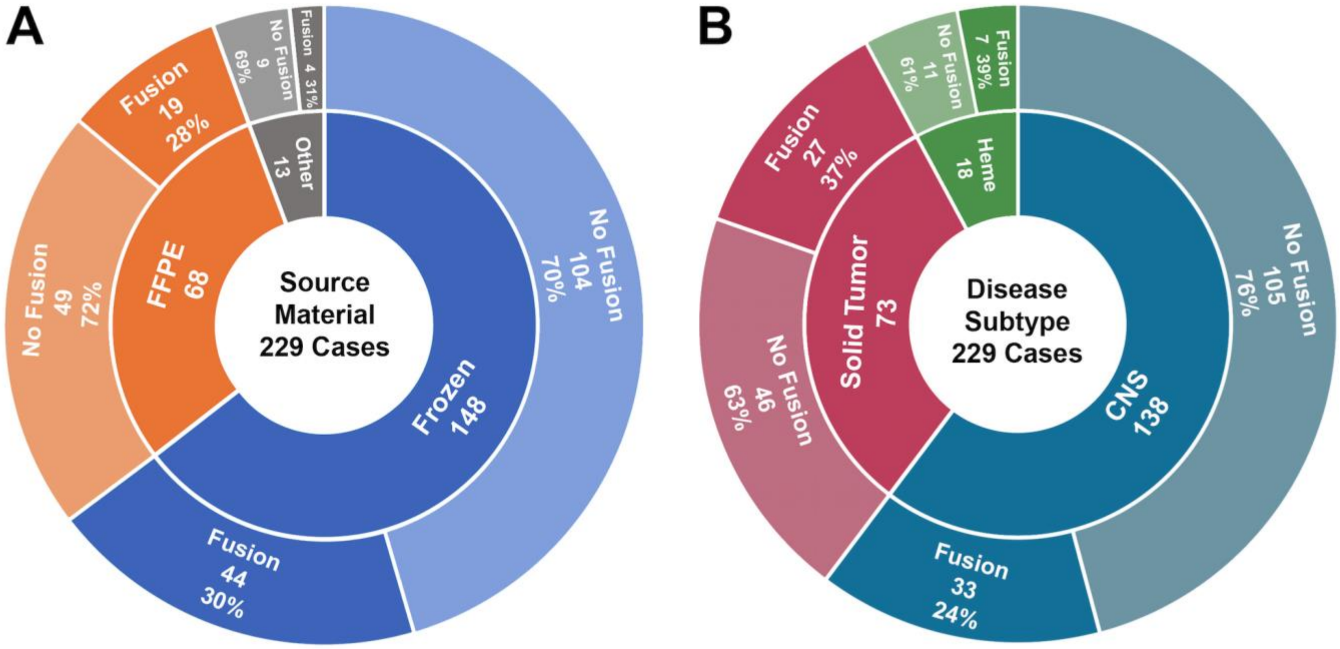

Figure S4. Fusions identified by source material and disease subtype in 229 pediatric cancer and hematologic malignancies. A) Independent of sample type, roughly 30% yield was identified across source material types, 44 clinically relevant fusions were identified out of 148 frozen specimens (30% yield), 19 were identified out of 68 FFPE specimens (28% yield), and 4 were identified out of 13 other specimens, which included blood, cerebral spinal fluid, and bone marrow (31% yield). **B)** The cohort is comprised of 138 CNS tumors, 73 solid tumors, and 18 hematologic malignancies. Clinically relevant fusions were identified at the following frequencies by subtype: 33 fusions identified out of 138 CNS specimens (24% yield), 27 fusions identified out of 73 solid tumors (37% yield), and 7 fusions identified out of 18 hematologic malignancies (39% yield). These results highlight the capability of the ensemble approach to identify fusions across pediatric cancer subtypes.

FIGURE S5

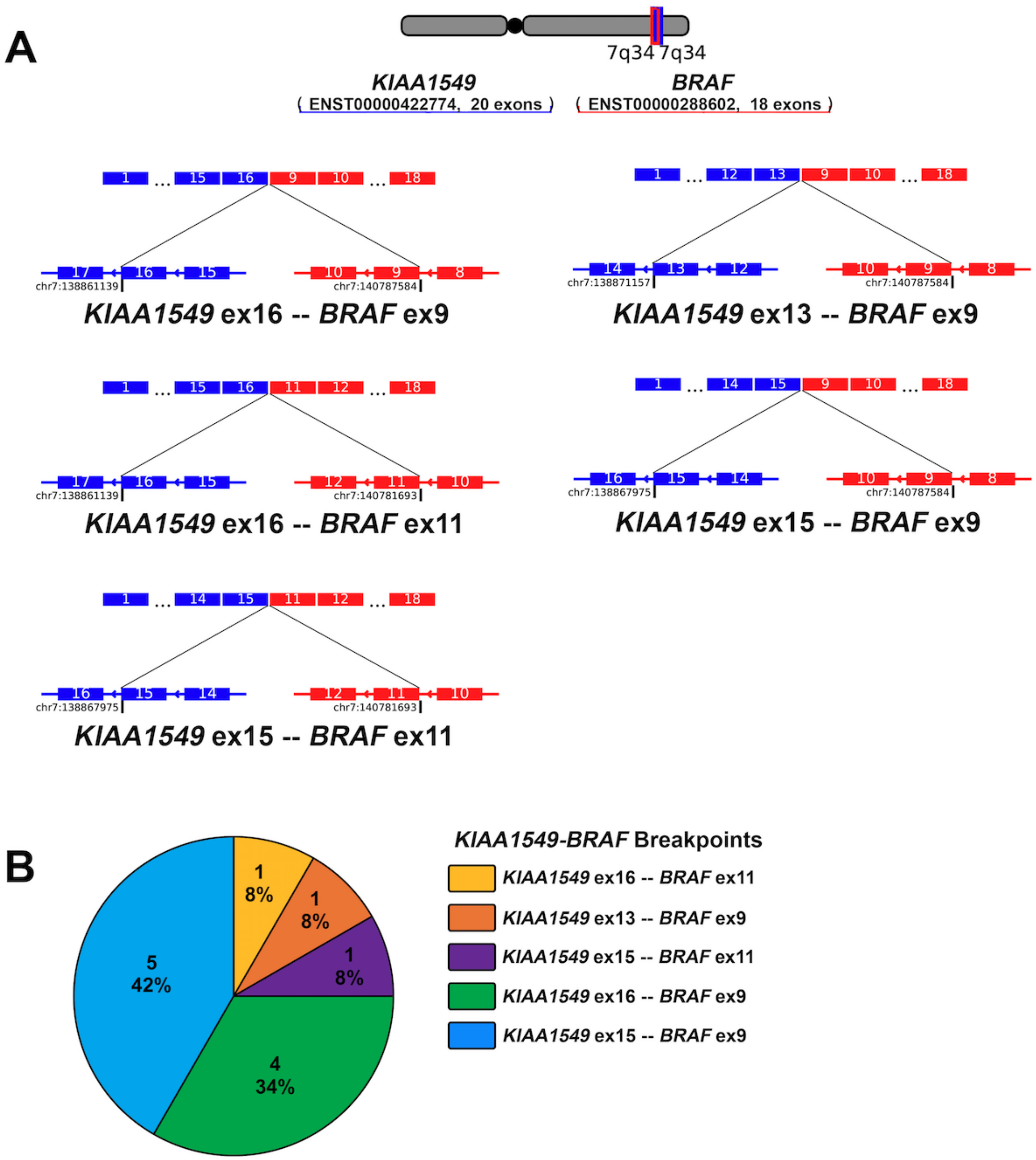

Figure S5. Breakdown of *KIAA1549-BRAF* fusions in NCH pediatric cancer and hematologic disease cohort. A) Five different fusion break points were identified in 12 cases, between *KIAA1549* (blue, ENST00000422774/NM_001164665) and *BRAF* (red, ENST00000288602/NM_004333). **B)** Composition of *KIAA1549-BRAF* fusion breakpoints in NCH cancer cohort.

FIGURE S6

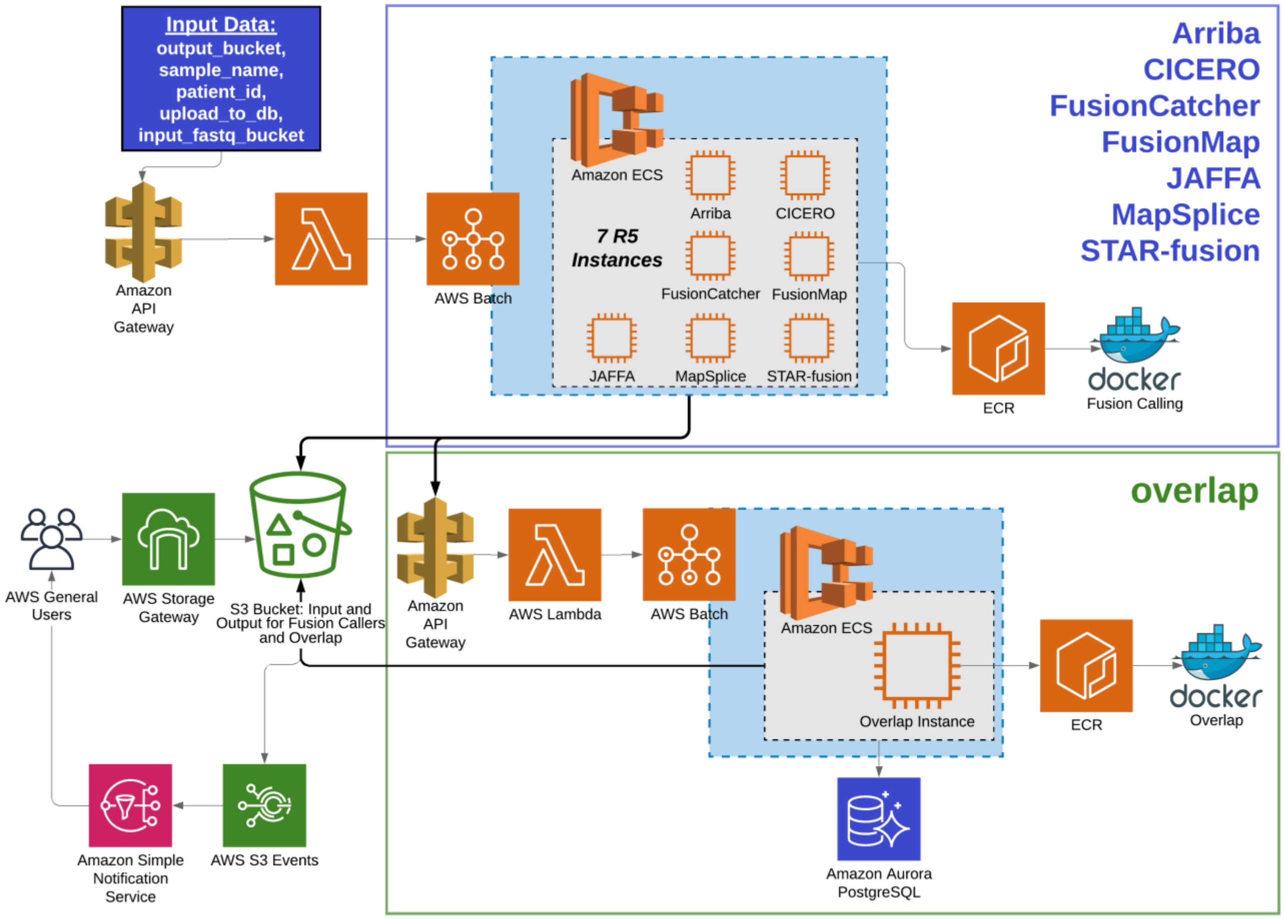

Figure S6. Ensemble fusion detection architecture diagram for serverless AWS implementation. Arguments are passed to Amazon API Gateway to invoke Lambda functions, kicking off AWS Batch runs for the Fusion Calling Docker image. Output is sent to an S3 bucket, invoking AWS Batch to kick off the overlap Docker image. The overlap Docker image queries and writes to the Aurora PostgreSQL database and performs all necessary filtering. Final results, including filtered, unfiltered, and singleton results are available in an S3 output bucket.

Figure S7

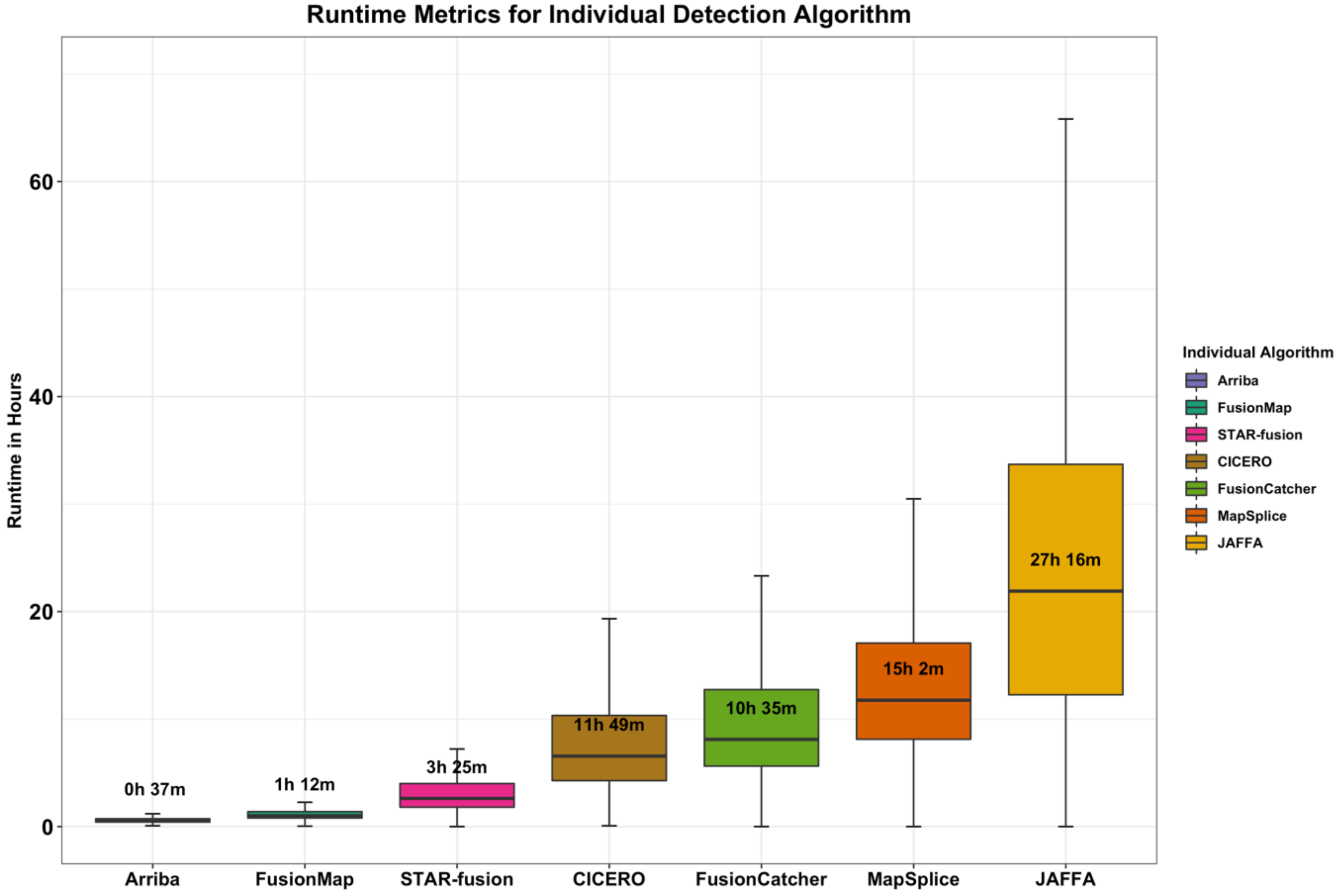

Figure S7. Averages and distribution of runtimes, in hours, for each fusion detection tool. Within the tested sample set, Arriba, FusionMap and STAR-Fusion tend to complete in a shorter period of time, even for samples with a large amount of sequencing data, while the remaining 4 tools take substantially longer to complete and have much greater variability in their runtimes. The data shown is from samples processed between January and November 2020, and includes 443 runs of Arriba, 865 runs of FusionMap, 851 runs of STAR-Fusion, 402 runs of CICERO, 827 runs of FusionCatcher, 836 runs of MapSplice and 965 runs of JAFFA. Data is ordered by median runtime, with average runtimes shown.

FIGURE S8

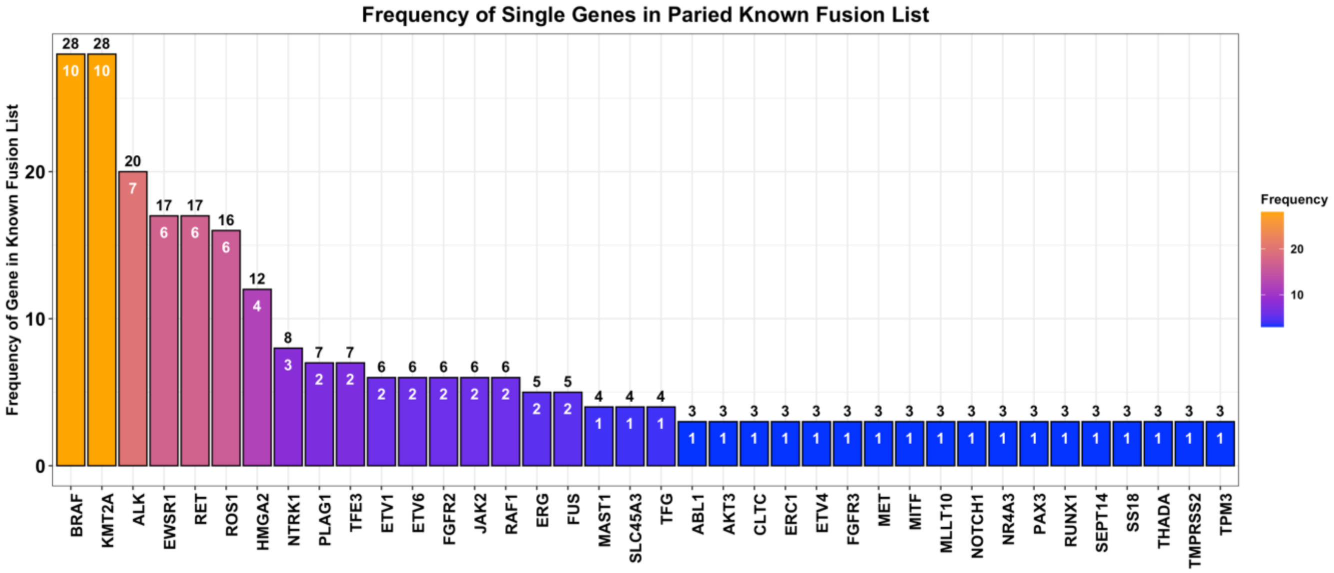

Figure S8. Frequency and associated pathogenic frequency scores of individual fusion partners in known fusion list. 38 genes are found commonly (≥3 times) as partners in the known fusion list. Frequency of gene as a partner is shown from high (orange, 28) to low (blue, 3), and associated scores in white are calculated as $Score = 10/(f_{max}-f)$, where f is the gene frequency and f_{max} is the maximum frequency observed.

Table S1

Unordered Fusion	COSMIC	TCGA
ABI1+KMT2A	+	
ABL1+BCR	+	+
ABL1+ETV6	+	
ABL1+NUP214	+	
ACE+KPNB1		+
ACSL3+ETV1	+	
ACTB+GLI1	+	
ACTN4+KMT2A	+	
AFDN+KMT2A	+	
AFF1+KMT2A	+	
AFF3+KMT2A	+	
AFF4+KMT2A	+	
AGGF1+RAF1		+
AGK+BRAF		+
AKAP13+RET		+
AKAP9+BRAF	+	
AKT3+ARHGAP30		+
AKT3+PLD5		+
AKT3+ZEB2		+
ALK+ATIC	+	
ALK+CARS	+	
ALK+CLTC	+	
ALK+DCTN1	+	
ALK+DDX6		+
ALK+EML4	+	+
ALK+GTF2IRD1		+
ALK+HIP1	+	
ALK+KIF5B	+	
ALK+KLC1	+	
ALK+MALAT1		+
ALK+MSN	+	
ALK+NPM1	+	
ALK+PPFIBP1	+	
ALK+RANBP2	+	
ALK+STRN	+	+
ALK+TFG	+	
ALK+TPM3	+	
ALK+TPM4	+	
ALK+VCL	+	
AP3B1+BRAF		+
ARHGAP26+CLDN18		+
ARHGAP26+KMT2A	+	
ARHGAP6+CLDN18		+
ARID1A+MAST2	+	
ASPSCR1+TFE3	+	
ATF1+EWSR1	+	
ATF1+FUS	+	
ATG7+BRAF		+
ATP1B4+CHST11		+
ATP5L+KMT2A		+
BAIAP2L1+FGFR3	+	
BCL2L11+BRAF		+
BCOR+ZC3H7B	+	
BCR+JAK2	+	
BICC1+FGFR2		+
BIRC6+LTBP1		+
BRAF+CCNY		+
BRAF+CEP89	+	
BRAF+CLCN6		+
BRAF+ERC1		+
BRAF+FAM114A2		+
BRAF+FAM131B	+	
BRAF+FCHSD1	+	
BRAF+GATM	+	
BRAF+GNAI1	+	
BRAF+HERPUD1	+	
BRAF+KIAA1549	+	
BRAF+LSM14A	+	
BRAF+MACF1		+
BRAF+MKRN1	+	+
BRAF+RNF130	+	
BRAF+RUNDC1		+
BRAF+SLC45A3	+	
BRAF+SND1	+	+
BRAF+SVOPL		+
BRAF+TAX1BP1		+
BRAF+TRIM24	+	
BRAF+ZC3HAV1		+
BRAF+ZSCAN30	+	
BRD3+NUTM1	+	
BRD4+NUTM1	+	
C11orf1+SIK3		+
C2CD2L+ZNF585B		+
C8orf34+MET		+
CADM2+MITF		+
CADM2+TFEB		+

Unordered Fusion	COSMIC	TCGA
CANT1+ETV4	+	
CAPZA2+MET		+
CBFA2T3+GLIS2	+	
CBFB+MYH11		+
CCDC186+FGFR2		+
CCDC6+RET	+	+
CCNB1IP1+HMGA2	+	
CD74+NRG1	+	
CD74+ROS1	+	+
CDH11+USP6	+	
CDKN2D+WDFY2	+	
CDX1+IRF2BP2	+	
CENPP+WNK2		+
CEP170B+KMT2A	+	
CHCHD7+PLAG1	+	
CIC+DUX4L1	+	
CIC+FOXO4	+	
CIPC+NGFR		+
CLCN6+RAF1	+	
CLIP1+ROS1	+	
CLTC+ROS1		+
CLTC+TFE3	+	
CNTNAP2+GRM8		+
COBL+SEPT14		+
COL1A1+PDGFB	+	
COL1A1+USP6	+	
COL1A2+PLAG1	+	
COL21A1+TFEB		+
COX6C+HMGA2	+	
CPSF4L+ERBB4		+
CREB1+EWSR1	+	
CREB3L1+FUS	+	
CREB3L2+FUS	+	
CREBBP+KAT6A		+
CREBBP+KMT2A	+	
CRTC1+MAML2	+	
CRTC3+MAML2	+	
CTNNB1+PLAG1	+	
CXorf67+MBTD1	+	
DDIT3+EWSR1	+	
DDIT3+FUS	+	
DDX20+TBX15		+
DHH+RHEBL1	+	
DHX33+NLRP1		+
DNAJB1+PRKACA	+	

Unordered Fusion	COSMIC	TCGA
DPM1+GRID1		+
DVL2+TFE3		+
EBF1+HMGA2	+	
EEFSEC+KMT2A	+	
EGFR+SEC61G		+
EGFR+SEPT14		+
EIF3E+RSPO2	+	
ELAVL3+FGFR3		+
ELL+KMT2A	+	+
EP300+KMT2A	+	
EPHA3+LCLAT1		+
EPHB1+MOBKL1B		+
EPS15+KMT2A	+	
ERBB2+MTSS1		+
ERBB4+EZR	+	
ERC1+RET	+	+
ERC1+ROS1	+	
ERG+EWSR1	+	
ERG+FUS	+	
ERG+NDRG1	+	
ERG+SLC45A3	+	+
ERG+TMPRSS2	+	+
ESRP1+RAF1	+	
ETV1+EWSR1	+	
ETV1+HNRNPA2B1	+	
ETV1+KLK2	+	
ETV1+SLC45A3	+	
ETV1+TMPRSS2	+	+
ETV4+EWSR1	+	
ETV4+TMPRSS2	+	+
ETV6+JAK2	+	
ETV6+MN1	+	
ETV6+NTRK3	+	+
ETV6+PDGFRB	+	
ETV6+RUNX1	+	
EWSR1+FEV	+	
EWSR1+FLI1	+	
EWSR1+MYB	+	
EWSR1+NFATC2	+	
EWSR1+NR4A3	+	
EWSR1+PBX1	+	
EWSR1+POU5F1	+	
EWSR1+WT1	+	
EWSR1+YY1	+	
EWSR1+ZNF384	+	

Unordered Fusion	COSMIC	TCGA
EWSR1+ZNF444	+	
EXOSC10+MTOR		+
EZR+ROS1	+	+
FGFR1+PLAG1	+	
FGFR1+TACC1	+	
FGFR2+FRK		+
FGFR2+OFD1		+
FGFR2+SHTN1		+
FGFR2+VCL		+
FGFR3+TACC3	+	+
FHIT+HMGA2	+	
FKBP15+RET		+
FLT3LG+RPS11		+
FLT4+LBH		+
FOXO1+PAX3	+	
FOXO1+PAX7	+	
FOXO3+KMT2A	+	
FOXP1+MITF		+
FRMD4B+MITF		+
GABBR2+NOTCH1	+	
GAS7+KMT2A	+	
GOLGA5+RET	+	
GOPC+ROS1	+	
GOSR1+ZNF207		+
GPBP1L1+MAST2	+	
H2AFX+WDR18		+
HACL1+RAF1	+	
HAS2+PLAG1	+	
HEY1+NCOA2	+	
HLA-A+ROS1	+	
HMGA2+LHFPL6	+	
HMGA2+LPP	+	
HMGA2+NFIB	+	
HMGA2+PCBP2		+
HMGA2+RAD51B	+	
HMGA2+SENP1		+
HMGA2+TSFM		+
HMGA2+WIF1	+	
HNF1B+NOTCH1		+
HOOK3+RET	+	
IGF2BP3+THADA		+
INSL3+JAK3		+
IRF2BP2+NTRK1		+
JAK2+PAX5	+	
JAK2+PCM1	+	

Unordered Fusion	COSMIC	TCGA
JAK2+SEC31A	+	
JAK2+SSBP2	+	
JAZF1+PHF1	+	
JAZF1+SUZ12	+	
KDM5A+NUP98	+	
KIAA1797+p16INK4		+
KIF5B+RET	+	
KMT2A+KNL1	+	
KMT2A+MLLT1	+	
KMT2A+MLLT10	+	+
KMT2A+MLLT11	+	
KMT2A+MLLT3	+	+
KMT2A+MLLT4		+
KMT2A+MLLT6	+	
KMT2A+SEPT2	+	
KMT2A+SEPT5	+	
KMT2A+SEPT6	+	
KMT2A+SEPT9	+	
KMT2A+TET1	+	
KSR1+TENM1		+
KTN1+RET	+	
LANCL2+SEPT14		+
LGR5+NUP107	+	
LIFR+PLAG1	+	
LMNA+NTRK1	+	
LRIG3+ROS1	+	
LRRC37B+NF1		+
LTK+UACA		+
MAML3+TCF4		+
MAML3+UBTF		+
MAST1+NFIX	+	
MAST1+NUP210		+
MAST1+TADA2A	+	
MAST1+ZNF700	+	
MEAF6+PHF1	+	
MECOM+RPN1		+
MECOM+RUNX1		+
MET+TFG		+
MLLT10+PICALM		+
MLLT10+PPP2R1B		+
MYB+NFIB	+	
MYO5A+ROS1	+	
NAB2+STAT6	+	
NACC2+NTRK2	+	
NBR1+WSB1		+

Unordered Fusion	COSMIC	TCGA
NCOA1+PAX3	+	
NCOA2+PAX3	+	
NCOA4+RET	+	+
NF1+RAB11FIP4		+
NONO+TFE3	+	
NOTCH1+SEC16A	+	
NR4A3+TAF15	+	
NR4A3+TFG	+	
NRG1+SLC3A2	+	
NSD1+NUP98		+
NTRK1+SQSTM1		+
NTRK1+SSBP2		+
NTRK1+TFG	+	+
NTRK1+TP53	+	
NTRK1+TPM3	+	
NTRK1+TPR	+	
NTRK2+QKI	+	
NTRK3+RBPMS		+
NUP214+SET	+	
NUTM2A+YWHAE	+	
NUTM2B+YWHAE	+	
OLR1+XIAP		+
PAX8+PPARG	+	+
PBX1+TCF3	+	
PCM1+RET	+	
PDCD6+TERT		+
PLAG1+TCEA1	+	
PML+RARA	+	+
PPFIBP1+ROS1	+	
PRCC+TFE3	+	+

Unordered Fusion	COSMIC	TCGA
PRKAR1A+RET	+	
PTPRK+RSPO3	+	
PWWP2A+ROS1	+	
RAF1+SRGAP3	+	
RAF1+TRAK1		+
RBM10+TFE3		+
RELCH+RET	+	
RET+SPECC1L		+
RET+TBL1XR1		+
RET+TRIM24	+	
RET+TRIM27	+	+
RET+TRIM33	+	+
RHOT1+TNKS		+
ROS1+SDC4	+	
ROS1+SHTN1	+	
ROS1+SLC34A2	+	+
ROS1+TPM3	+	
ROS1+ZCCHC8	+	
RPS2P32+THADA		+
RUNX1+RUNX1T1	+	+
SFPQ+TFE3	+	+
SLC16A14+TRIP12		+
SS18+SSX1	+	+
SS18+SSX2	+	+
SS18+SSX4	+	
STIL+TAL1	+	
TBL1XR1+TP63	+	
TERT+TRIO		+
TG+THADA		+

Table S1. Inclusion of known pathogenic fusion partners in a known fusion list to prevent over-filtering of known fusions by ensemble pipeline. The known fusion list includes a total of 325 fusion partners, 191 of which were identified by COSMIC with at least 1 sample tested for the fusion, 104 were identified as known fusions by The Cancer Genome Atlas (TCGA) as reported by Gao et al., 2018, and 30 were identified by both [27, 57] Fusions commonly found in normal tissue have been removed from this list.

TABLE S2

RNA Fusion	Thermo Fisher Unique Assay ID	Digital PCR copies/µL Member 1
EML4-ALK	Hs04419883_ft	18700
KIF5B-RET	Hs04396834_ft	25147
NCOA4-RET	Hs04396875_ft	65813
CD74-ROS1	Hs04396897_ft	21260
SLC34A-ROS1	Hs04396942_ft	35120
TPM3-NTRK1	Hs03024839_ft	29493
FGFR3-BAIAP2L1	Hs04396791_ft	31120
PAX8-PPARG1	Hs04396714_ft	66027
FGFR3-TACC3	Hs04421327_ft	18480
ETV6-NTRK3	Hs03024415_ft	74400
LMNA-NTRK	Hs04421343_ft	32800
SLC45A3-BRAF	Hs04396966_ft	70720
TMPRSS2-ERG	AIHSQLA (custom)	23280
EGFR-SEPT14	AJCSWD6(custom)	50907

Table S2. Digital PCR Results for Undiluted Fusion RNA v2 Dilution Panel RNA. Results are given as copies of fusion RNA per µL of volume.

TABLE S3

RNA Fusion	Thermo Fisher Unique Assay ID	Digital PCR copies/µL Member 1
EML4-ALK	Hs04419883_ft	1347
KIF5B-RET	Hs04396834_ft	539
NCOA4-RET	Hs04396875_ft	797
CD74-ROS1	Hs04396897_ft	891
SLC34A-ROS1	Hs04396942_ft	820
TPM3-NTRK1	Hs03024839_ft	916
FGFR3-BAIAP2L1	Hs04396791_ft	617
PAX8-PPARG1	Hs04396714_ft	1608
FGFR3-TACC3	Hs04421327_ft	1291
ETV6-NTRK3	Hs03024415_ft	879
LMNA-NTRK	Hs04421343_ft	643
SLC45A3-BRAF	Hs04396966_ft	804
TMPRSS2-ERG	AIHSQLA (custom)	605
EGFR-SEPT14	AJCSWD6(custom)	829

Table S3. Digital PCR Results for Seraseq Fusion RNA v3 (before dilution). Results are given as copies of fusion RNA per µL of volume [53].

TABLE S4 – PLEASE SEE ADDITIONAL FILE 2

Supplemental Materials Table S4. Precision and sensitivity of ensemble approach in Seraseq fusion reagent RNA-seq data. Row highlighted blue is the average of undiluted (neat) sample duplicates, which is represented in **Figure 1B**. Rows highlighted green are averages per dilution or negative control triplicates (Additional File 2).

Table S5

	EnFusion ≥ 2	EnFusion ≥ 3	STAR-fusion	FusionMap	FusionCatcher	JAFFA	Map	Arriba	CICERO
Minimum	1	0	14	17	244	450	18	10	685
25% Percentile	3	0.25	19	22	815	1151	28	20	880
Median	8	2.5	26	28	1272	1427	37	25	1046
75% Percentile	17	13	30	37	1448	1637	42	34	1186
Maximum	21	14	39	49	2005	2086	58	39	1488
Mean	9.6	6.1	25	29	1166	1368	36	26	1043
Std. Deviation	7	6.1	7.2	9.1	465	408	9.6	8.5	208
Std. Error of Mean	1.6	1.4	1.6	2	104	91	2.1	1.9	47
Lower 95% CI of mean	6.3	3.2	22	25	948	1177	31	22	945
Upper 95% CI of mean	13	8.9	29	34	1384	1559	40	30	1140
Caller vs. EnFusion ≥2 Callers p value	-	2.34E-07	3.69E-10	7.85E-10	1.03E-08	3.42E-10	3.19E-08	2.92E-09	3.53E-14
Caller vs. EnFusion ≥3 Callers p value	2.34E-07	-	1.77E-12	3.87E-11	1.00E-08	3.34E-10	2.89E-09	8.63E-11	3.39E-14

Table S5. Individual caller statistics for number of total fusions identified in SeraCare Seraseq fusion reagent RNA-seq data.

Data from ensemble approach with filtering and whitelist shown. Paired student t tests performed on raw values.

TABLE S6

	Fusion	Initial Identification Method	Arriba	JAFFA	Fusion Map	Map splice	STAR-fusion	CICERO	Fusion Catcher	Total Callers	Full Karyotype, Centrally Reviewed
1	*MLLT10-PICALM*	Karyotyping	1	1	1	1	1	1	1	7	46,XY,?del(1)(p36.1), t(10;11)(p13;q21) [15]/46,XY[5]
2	*RUNX1-RUNX1T1*	Karyotyping	1	0	0	0	1	1	1	4	46,XX,t(8;21)(q22;q22)[20]
3	*CBFB-MYH11*	Karyotyping	1	1	1	1	1	0	1	6	46,XX,inv(16)(p13q22)[12]
4	*CBFB-MYH11*	Karyotyping	1	1	1	1	1	1	1	7	46,XY,inv(16)(p13q22)[18]/46,XY [2]
5	*CBFB-MYH11*	Karyotyping	1	1	1	1	1	1	1	7	46,XY,inv(16)(p13.1q22)[20]
6	*RUNX1-RUNX1T1*	Karyotyping	0	1	1	1	1	0	1	5	46,XY,t(8;21)(q22;q22),del(9)(q12q22)[10]/45, X,-Y,t(8;21) (q22;q22) [3]/45,X,-Y,t(8;21) (q22; q22), del(9)(q12q22)[8]
7	*CBFB-MYH11*	Karyotyping	0	1	0	0	0	1	1	3	46,XY,inv(16)(p13q22)[25]
8	*CBFB-MYH11*	Karyotyping	1	1	1	1	1	1	1	7	46,XX,inv(16)(p13q22)[15]/46,XX [6]
9	*KMT2A-MLLT3*	Karyotyping	0	1	0	0	1	0	1	3	46,XX,t(9;11)(p21;q23)[20]
10	*CBFB-MYH11*	EnFusion	1	1	1	1	1	1	1	7	46,XX,inv(4)(p16?q31.1),add(14)(p11),-16,+mar[20]
11	*KMT2A-MLLT10*	EnFusion	1	1	1	0	1	1	1	6	46,XY,add(10)(p13),del(11)(q11)[12]/46,idem,add(1)(p36)[8]
		**Individual Caller Sensitivity**	**72.7%**	**90.9%**	**72.7%**	**63.6%**	**90.9%**	**72.7%**	**100.0%**	-	

Table S6. Benchmarking of the ensemble approach on an external acute myeloid leukemia dataset identifies 100% sensitivity. Adult acute myeloid leukemia (AML) samples (n=11) from an external cohort were utilized, individual caller outputs are shown, where 1=fusion identified and 0=fusion not found. Fusions 1-9 were originally identified through karyotyping and expert review by a cytogeneticist in a clinical laboratory. Fusions 10-11 were initially identified by EnFusion, and then confirmed through reanalysis of the original karyotyping results by a cytogeneticist in a clinical laboratory. Full centrally reviewed karyotyping results are shown.

TABLE S7

Dataset	Ensemble (≥2 callers)	Ensemble (≥3callers)	STAR-fusion	FusionMap	FusionCatcher	JAFFA	MapSplice	Arriba	CICERO
BEERS 1	1	1	5	6	158	3	5	206	285
BEERS 2	0	0	1	0	19	0	1	0	10
BEERS 3	0	0	2	1	10	0	2	0	6
Total false positives	1	1	8	7	187	3	8	206	301

Table S7. EnFusion benchmarking studies utilizing negative control data highlight low false discovery rate. Three synthetic data sets were generated using Benchmarker for Evaluating the Effectiveness of RNA-Seq Software (BEERS), and results from the ensemble approach as well as individual tools are shown. The BEERS1 dataset was previously generated and described by Davidson et al., *Genome Med* 2015. The fewest number of false positives (n=1) was identified using the ensemble approach.

TABLE S8

	EnFusion ≥ 2	EnFusion ≥ 3	STAR-fusion	FusionMap	FusionCatcher	JAFFA	Map	Arriba	CICERO
Minimum	0	0	6	0	7	1	0	0	27
25% Percentile	3.5	1	35	16	306	459	16	12	1156
Median	8	2	47	25	551	766	29	29	1604
75% Percentile	18	5	80	38	1082	1220	49	73	2136
Maximum	709	72	1696	695	38726	16352	175	515	8634
Mean	28	4	71	34	1554	1134	37	54	1909
Std. Deviation	77	7.9	119	51	4347	1542	31	68	1280
Std. Error of Mean	5.1	0.52	7.9	3.4	287	102	2.1	4.5	85
Lower 95% CI of mean	18	3	56	28	988	934	33	45	1742
Upper 95% CI of mean	38	5.1	87	41	2120	1335	41	63	2075
Caller vs. EnFusion ≥2 Callers p value	-	3.43E-06	3.85E-10	7.25E-02 N.S.	1.76E-07	1.83E-23	6.25E-02 N.S.	7.92E-05	3.92E-61
Caller vs. EnFusion ≥3 Callers p value	3.43E-06	-	1.08E-15	3.42E-17	1.70E-07	3.07E-23	2.14E-42	1.96E-23	6.66E-60

Table S8. Individual caller statistics for number of total fusions identified in pediatric cancer and hematologic disease cohort data. Data from ensemble approach with filtering and whitelist shown. Paired Student t tests performed on raw values, N.S. = not significant.

TABLE S9 – PLEASE SEE ADDITIONAL FILE 3

Table S9. Fusions identified in pediatric cancer and hematologic disease cohort (Additional File 3). [1]Clinical confirmation methods include testing performed in a CAP-accredited, CLIA-validated clinical laboratory.

TABLE S10

CaseID	ITD Identified by CICERO	Orthogonal Confirmation
**IGMCH0053**	EGFR	Yes (Archer FusionPlex panel)
**IGMCH0113**	FGFR1	Yes (Archer FusionPlex panel; RT-PCR followed by Sanger sequencing)
**IGMCH0130**	EGFR	Yes (Archer FusionPlex panel)
**IGMCH0144**	FGFR1	Did not confirm by Archer FusionPlex panel
**IGMCH0248**	PDGFRA	Did not confirm by Archer FusionPlex panel
**IGMCH0252**	FLT3	Not tested
**JL_17015_M17-2670**	FGFR1	Yes (Archer FusionPlex panel; RT-PCR followed by Sanger sequencing)

Table S10. Internal Tandem Duplications (ITD) identified by CICERO in NCH Cohort. 7 clinically relevant ITDs were identified within the cohort, 4 of which were confirmed using orthogonal assays. These results highlight the benefit of the overlap approach aggregating and utilizing individual pipeline outputs.

TABLE S11

Gene	Description	Frequency	scores
BRAF	serine/threonine-protein kinase	28	10
KMT2A	methyltransferase	28	10
ALK	tyrosine kinase	20	7
EWSR1	transcriptional regulator	17	6
RET	tyrosine kinase	17	6
ROS1	DNA glycosylase/lyase	16	6
HMGA2	transcriptional regulator	12	4
NTRK1	tyrosine kinase	8	3
PLAG1	transcription factor	7	2
TFE3	transcription factor	7	2
ETV1	transcriptional activator	6	2
ETV6	transcriptional repressor	6	2
FGFR2	tyrosine kinase	6	2
JAK2	tyrosine kinase	6	2
RAF1	serine/threonine-protein kinase	6	2
ERG	potassium channel	5	2
FUS	transcriptional regulator	5	2
MAST1	microtubule protein	4	1
SLC45A3	solute carrier	4	1
TFG	Oncogene	4	1
ABL1	Oncogene	3	1
AKT3	endoplasmic reticulum transport	3	1
CLTC	kinetochore stabilization	3	1
ERC1	regulatory subunit of IKK	3	1
ETV4	transcriptional activator	3	1
FGFR3	transcriptional activator	3	1
MET	tyrosine kinase	3	1
MITF	transcription factor	3	1
MLLT10	transcriptional regulator	3	1
NOTCH1	transcriptional regulator	3	1
NR4A3	transcriptional activator	3	1
PAX3	transcriptional activator	3	1
RUNX1	transcriptional regulator	3	1
SEPT14	cytoskeletal GTPase	3	1
SS18	transcriptional activator	3	1
THADA	adenoma associated	3	1
TMPRSS2	protease	3	1
TPM3	actin filament associated	3	1

Table S11. Commonly occurring fusion partners in known fusion list are utilized as pathogenicity predictor using a score based on gene partner frequency. 38 genes are present 3 or more times as partners on the known fusion list (see **ADDITIONAL FILE 1: TABLE S1** for known fusion list). Pathogenic frequency scores were developed to predict pathogenicity of a novel, or not well described, fusion based on gene partners known to be common in well described pathogenic fusions. The pathogenic frequency score ranges from 10 (most frequent) to 1 (least frequent, but present at least 3 times): *Score = 10/(28-gene frequency)*. The annotations that are provided in the ensemble output if one of the 38 common pathogenic gene partners is found, are as follows: designation as a known pathogenic gene partner, inclusion of the frequency score (1-10), and a description of the gene type based on UniProt information.
